# Innovation in Permanent Pacemaker’s Implantation Technique: Trans-Axillary Approach

**DOI:** 10.7759/cureus.14436

**Published:** 2021-04-12

**Authors:** Bakhtawar Shah, Muhammad Amir Niaz, Shahab Saidullah, Farrukh Zaman, Hassan Mumtaz, Aamir Ghazanfar

**Affiliations:** 1 Division of Clinical Cardiac Electrophysiology, Department of Cardiology, Hayatabad Medical Complex, Peshawar, PAK; 2 Interventional Cardiology, Ali Medical Center, Islamabad, PAK; 3 Clinical Cardiac Electrophysiology, Pakistan Instituite of Medical Sciences, Islamabad, PAK; 4 Diabetes and Endocrinology, Kahutta Research Laboratory (KRL) Hospital, Islamabad, PAK; 5 Urology, Guys and St Thomas Hospital, London, GBR; 6 General Medicine, Surrey Docks Health Center, London, GBR; 7 Surgery, Kahutta Research Laboratory (KRL) Hospital, Islamabad, PAK; 8 Vascular Surgery, Kahutta Research Laboratory (KRL) Hospital, Islamabad, PAK

**Keywords:** permanent pacemakers, venogram, axillary vein, lead, erosion, implantation

## Abstract

Introduction: Permanent pacemakers’ (PPM) implantation is an integral part of electrophysiology and general cardiology. The implantation technique has evolved a lot since the first implantation. Several innovations have been undertaken to improve the effectiveness, life of the transplant, and patient outcomes. In this study, we introduced a new implantation technique to improve the procedure and possibly reduce the rate of complication.

Methods: This study was conducted from January 2016 to February 2017 in Hayatabad Medical Complex, Peshawar. Patients destined for implantation of PPM based on a clinical treatment plan, after proper explanation of the procedure, were brought to the catheterization laboratory. Venogram of the upper limb performed. Patients were scrubbed and draped. The axillary vein was approached via the Seldinger technique. About 2 to 3 cm superolateral to the puncture site, a skin incision was made and subcutaneous pocket constructed, and a guidewire external end was pulled in from inside the pocket keeping the venous end at the place. Subsequently, in a routine way, lead was placed, secured and the wound was closed in layers.

Results: A total of 690 PPM were implanted under the study. About 290 devices were implanted in the conventional way and 380 devices via the trans-axillary approach. The mean implantation time was less than 30 minutes via the trans-axillary approach. Immediate and delayed complications of the procedure were minimal.

Conclusion: Trans-axillary approach holds some significant advantages over the conventional technique. The subcutaneous pocket and venous puncture successfully reduce the burden of foreign material, minimize the tension on the wound, shorten implantation time and reduce the chances of erosion of the device.

## Introduction

There is a linear relationship between cardiac health with the increase in average life expectancy all over the world [1]. Coronary artery diseases (CAD) are emerging as a new epidemic of our present era, which is not only increasing the burden on the catheterization laboratories but also leading to the increased volume of the atrioventricular block (AV block) of different categories. Certain degenerative diseases lead to the heart’s conduction system block [2,3]. Besides CAD and degenerative conditions, congenital cardiac anomalies and cardiac procedures for congenital anomalies or acquired conditions contribute significantly to the symptomatic conducting abnormalities which have increased the implantation volume of permanent pacemakers (PPM), automated implantable cardioverter defibrillators (AICD), and cardiac resynchronization therapy (CRT) devices [4-7]. All these devices are implanted more or less in the same way mostly under local anesthesia. With time technologies evolve and there is a revolution from big abdominal devices to very small devices, and even leadless devices [8,9]. The lead technology improved a lot as well. Recently, thin flexible bipolar leads are available that are very well accommodated in the subcutaneous pocket under the device [10].

Not only the technology but the methods of implantation, prevention of infection with good antibiotic cover, and modern sterilization techniques have reduced the complication of implantation many folds [11-13]. In our settings, the devices are implanted by the Seldinger technique through the axillary vein [14] which is less traumatic and very less time-consuming and needs minimal surgical skill as compared to the cephalic vein [15] approach that needs good surgical skills, and subclavian vein [14], where the chance of pneumothorax increases many folds. But despite this entire revolution one big problem still exists and that is lead erosion and wound dehiscence [16,17] which may occur very early or late at any time after implantation.

We introduce an innovative technique to optimize and hasten the procedure that will possibly reduce the chances of wound dehiscence.

## Materials and methods

All those patients who presented to our unit from January 2016 to February 2017 in Hayatabad Medical Complex, Peshawar, due to any cause for implantation of PPMs were admitted and informed consent was obtained. Baseline investigation including renal function test (RFTs), serum electrolytes, blood sugar (RBS), virology, full blood count (FBC), liver function tests (LFTs), and cardiac enzymes were analyzed. Male patients’ chests were shaved at night. Both male and female patients’ chests were painted at night. Night sedation with 3 mg bromazepam orally was administered. Patients were nil by oral when they were brought to the catheterization laboratory. An intravenous cannula was passed on both upper limbs. Pre-medication with intravenous (IV) dimenhydrinate, injection nalbuphine, and at the time, injection Midazolam was given if the patient was restless or uncooperative at the time of implantation. It is our usual practice to give IV antibiotics half an hour before surgery, and a second dose during surgery or just at the end of the procedure if implantation time is less than 30 minutes.

After the patient was scrubbed and draped, a venogram of the axillary vein on the side of implantation was performed. Lidocaine 2% infiltrated at the site of implantation. Axillary vein puncture was done by Seldinger technique before skin incision and guidewire passed. Two- to three-centimeter superolateral to the puncture site, a skin incision was made, and a self-retaining retractor was applied. Using artery forceps with blunt dissection, the subcutaneous tissue separated till the deep fascia was visible. The flap of the skin was left with tooth forceps and dissection extended anteromedially to construct the pocket for the device. Guidewires were localized inside the pocket. Once the guidewire came into the site, the outer end of the wires was pulled in from inside the pocket. So, the outer end came in and came out through the incision while the venous end was in place. This brought the venous prick inside the pocket for the device.

Then, in the usual way, the sheath was passed over the wire. Wire with dilator was pulled out. PPM lead was positioned in the right ventricle and right atrium. The leads were stabilized with muscle using 1/0 non-absorbable sutures. The pocket for the device was irrigated with gentamicin to remove any residual clots. The pulse generator was attached with lead. The device was inserted inside the pocket and a safety stitch was placed to hang the device inside the pocket with non-absorbable sutures. The rest of the lead which remained outside the venous prick was folded and placed below the pulse generator. Now, the whole hardware is inside the pocket, and no need to bury the lead in the muscles. The pocket mouth was closed with a single absorbable suture. Subcutaneous tissue was approximated with absorbable suture and the skin was closed with interrupted non-absorbable sutures. This technique placed all the hardware away from the suture line.

## Results

A total of 670 PPM was implanted in the study period. The results are tabulated in Table 1. About 290 devices were implanted in the conventional way and 380 on the new method. There were 350 male and 320 female participants in the study. The mean age of the patients was 60.47±16.35 years in the study group. Only 2.6% of patients were below the age of 20 years and 0.1% below the age of 10 years. The minimum age in the study was 10 years and a maximum of 100 years. There were 93% patients from Pakistan and 7% from Afghanistan. The total implantation time was 30-45 minutes on average in the new way of implantation, while it was more than 60 minutes in the conventional way (p-value = 0.000). However, total fluoroscopy time remained from five to eight minutes in both types of procedures. There were 500 dual-chamber pacemakers and 170 single-chamber pacemakers. In four cases, the devices were implanted from the right side due to persistent left side SVC. One case had stenosed veins on both sides in the implantation area, so the device was implanted through SVC after thoracotomy under general anesthesia by a cardiothoracic surgeon.

**Table 1:** Demographic data of the patients. SVC: superior vena cava.

Method of procedure	Conventional	New method	P-value
Number	290 (43.28%)	380 (56.71%)	
Male	140 (20.89%)	210 (31.34%)	
Female	150 (22.38%)	170 (25.37%)	
Time of implantation	30-40 minutes	60-80 minutes	0.000
Dual chambers devices	210 (31.34)	300 (44.77%)	
Single chamber devices	80 (11.94%)	80 (11.94%)	
Persistent left SVC	1 (0.14%)	3 (o.44%)	
Pneumothorax	2 (0.29%)	10 (1.49%)	0.000
Lead erosion	0	04 (0.59%)	0.000
Silk suture	5	5	
Vicryl 1/0	2-3	7-10	0.000
Proline 1/0	3-4	3-4	

The complications in both procedures were different. There were two cases of pneumothorax in the new method during the procedure while 10 cases were complicated by pneumothorax in the conventional way of implantation. This difference was statistically significant (p-value = 0.000). On follow-up, four patients presented with lead erosion who was operated on by the conventional way of implantation, but no such case was reported in our new method of implantation.

There were five silk sutures in dual-chamber pacemakers for stabilization of lead and three silk sutures in single chambers of devices in both types of procedure. Vicryl 1/0 was used to close the wound in layers. There were two to three sutures of Vicryl 1/0 in the new technique, but about 7-10 sutures were needed in the conventional method (p-value = 0.000). The skin was closed with proline 1/0 interrupted three to four sutures in both cases.

## Discussion

Permanent pacemaker implantation is a common interventional procedure around the globe. There are certain complications of the procedure. Lead erosion is a notorious complication in the process of device implantation [17]. It not only leads to increased morbidity of the patients but also it is responsible for increasing the economic burden on index patient and society. Once the device is exposed it needs to be explanted along with the pulse generator [17] and the lead explanation is a very cumbersome procedure most of the time [18]. In a third-world country, most of the time, the public sector hospital lacks the adequate facility of explantation. Explantation also demands highly experienced operators for the success of the procedure. So, it is crucial to devise and adopt methods to minimize the chances of lead erosion.

Infection, as a result of surgical wounds, is a serious problem in developing countries [19] which can cause lead erosion. But implantation techniques and operator experience are also important factors that may be responsible for lead erosion [17]. In the past practice, after the skin incision and dissecting the subcutaneous tissue with an artery forceps till the deep fascia is visible on the underlying muscle, we used to construct the subcutaneous pocket for the pulse generator and then picked the axillary vein with the Seldinger technique. In this way of implantation, the lead came in the center of the wound, and then after stabilizing the lead with the underlying muscle it needs to be buried in the muscle by taking a purse-string suture around the lead’s sleeve. In this whole process, there is the use of extra suture materials and tension on the healthy muscle which may lead to necrosis of the muscle [20] and maybe a nidus for infection on one side, and the sleeve which is lying just below the incision line may cause wound dehiscence [17,21].

Some centers still approach the cephalic vein [15] and use a cut-down procedure that requires surgical skill and is time-consuming. Another approach to the subclavian vein [14], though this approach is very easy, again the lead comes in the line of wound and taking muscle around the lead sleeve will create undue tension on the healthy tissue and may cause necrosis [20,22] which will lead to infection and erosion [17] on one hand and the lead is prone to Subclavian Lead Crush Syndrome [22]. On the other hand, with the subclavian approach, the chance of pneumothorax is very high [23].

Therefore, we adopted a different approach to take undue tension away from the suture line. We puncture medial and inferior to the incision line, as explained in the methodology, so it takes everything inside the pocket made for the pulse generator, away from the line of incision and skin sutures. So, there is no tension on the skin stitching line on one hand and there is no need to bury the lead in the muscle fold on the other hand. So, the whole assembly comes inside the pocket. In this way, the approximation of the subcutaneous tissue is very clean without any stretching on the subcutaneous tissue. There is nothing in the incision line and no tension on the skin suture. If we take care of the infection, there is almost no chance of wound dehiscence or lead erosion in the early or late post-operation period. This method is very safe, effective, and convenient for the elderly who are very skinny. So, there is less chance of device erosion.

Sutures are the backbone of the tensile strength. But increasing the suture materials will increase the chance of infection. The wound gains its tensile strength over time through healing [24]. Tissue takes about three months to gain maximum strength and about six months for full strength [25]. Vicryl’s maximum tensile strength is for 14 days, but it takes about 70 days to get absorbed completely [26]. This extended period increases the risk of infection rather than of any benefit to the tensile strength of the wound. Similarly, increasing the number of suture materials increases the chances of infection without any benefit to the strength of the wound. The amount of suture material is significantly reduced with this new technique.

However, this method is not convenient in very obese people where a lot of fatty tissue will not allow the needle to reach the axillary vein through the intact skin. Therefore, in this individual, prior incision, separation, and dissection of subcutaneous tissue will only allow the needle to reach the target. The method may be employed even in obese people. So, if the needle meets the vein, the rest of the procedure becomes very easy and less time-consuming.

To sum up, we emphasize that pacemaker implantation is an art that has a lot of room for improvement and refinement. Despite different complication rates has been reported with different methods, it is the operator who can choose according to the situation, his experience and convenience, and body habitus of the patients.

## Conclusions

We found this method less time-consuming. There is almost no chance of pneumothorax since the axillary vein is outside the lung field with minimum tension on the suture line, so the natural process of healing is hastened. The healthy muscle remains undisturbed, so there is less postoperative discomfort for the patient and minimal use of external suture materials, and fewer chances of infection.
